# (Heptanedioato-κ^2^ *O*,*O*′)bis­(1,10-phenanthroline-κ^2^ *N*,*N*′)zinc(II) hexa­hydrate

**DOI:** 10.1107/S1600536808006004

**Published:** 2008-03-07

**Authors:** Jian-Li Lin, Yuan-Yuan Wang

**Affiliations:** a State Key Laboratory, Base of Novel Functional Materials and Preparation Science, Institute of Solid Materials Chemistry, Faculty of Materials Science and Chemical Engineering, Ningbo University, Ningbo 315211, People’s Republic of China

## Abstract

In the crystal structure of the title compound, [Zn(C_7_H_10_O_4_)(C_12_H_8_N_2_)_2_]·6H_2_O, the Zn^II^ atom is coordinated by two carboxyl­ate O atoms of a mono-bidentate chelating pimelate anion (pimelic acid is hepta­nedioic acid) and four N atoms of two phenanthroline ligands, forming a considerably distorted octa­hedral ZnN_4_O_2_ coordination geometry. The complexes are assembled into a three-dimensional network *via* C—H⋯O, C—H⋯π and π–π inter­actions. The mean inter­planar distance between adjacent phenanthroline ligands is 3.399 (2) Å.

## Related literature

For related literature, see: Ge & Zheng (2005 ▶); Wei *et al.* (2002 ▶); Zheng (2004 ▶); Zheng, Kong & Chen (2003 ▶); Zheng, Lin & Kong (2003 ▶); Zheng *et al.* (2001 ▶, 2002 ▶); Zheng & Ying (2004 ▶).
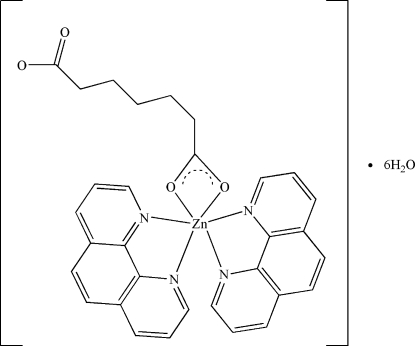


## Experimental

### 

#### Crystal data

[Zn(C_7_H_10_O_4_)(C_12_H_8_N_2_)_2_]·6H_2_O
*M* *_r_* = 692.02
Monoclinic, 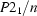

*a* = 9.2050 (18) Å
*b* = 21.241 (4) Å
*c* = 16.598 (3) Å
β = 96.48 (3)°
*V* = 3224.6 (11) Å^3^
*Z* = 4
Mo *K*α radiation
μ = 0.82 mm^−1^
*T* = 296 (2) K
0.43 × 0.26 × 0.22 mm

#### Data collection

Bruker P4 diffractometer
Absorption correction: ψ scan (North *et al*., 1968 ▶) *T*_min_ = 0.697, *T*_max_ = 0.834
9226 measured reflections
7393 independent reflections
3956 reflections with *I* > 2σ(*I*)
*R*_int_ = 0.043
3 standard reflections every 97 reflections intensity decay: no

#### Refinement

*R*[*F* ^2^ > 2σ(*F* ^2^)] = 0.059
*wR*(*F* ^2^) = 0.117
*S* = 1.01
7393 reflections
453 parameters
18 restraints
H atoms treated by a mixture of independent and constrained refinement
Δρ_max_ = 0.34 e Å^−3^
Δρ_min_ = −0.29 e Å^−3^

### 

Data collection: *XSCANS* (Siemens, 1996 ▶); cell refinement: *XSCANS*; data reduction: *XSCANS*; program(s) used to solve structure: *SHELXS97* (Sheldrick, 2008 ▶); program(s) used to refine structure: *SHELXL97* (Sheldrick, 2008 ▶); molecular graphics: *SHELXTL* (Sheldrick, 2008 ▶); software used to prepare material for publication: *SHELXTL*.

## Supplementary Material

Crystal structure: contains datablocks global, I. DOI: 10.1107/S1600536808006004/is2271sup1.cif

Structure factors: contains datablocks I. DOI: 10.1107/S1600536808006004/is2271Isup2.hkl

Additional supplementary materials:  crystallographic information; 3D view; checkCIF report

## Figures and Tables

**Table:** 

Zn—N4	2.128 (3)
Zn—N3	2.129 (3)
Zn—N2	2.148 (3)
Zn—N1	2.167 (3)
Zn—O1	2.178 (3)
Zn—O2	2.224 (3)

**Table:** 

N4—Zn—N3	78.26 (11)
N4—Zn—N2	99.28 (11)
N3—Zn—N2	168.21 (11)
N4—Zn—N1	109.01 (11)
N3—Zn—N1	92.26 (11)
N2—Zn—N1	77.55 (11)
N4—Zn—O1	102.80 (10)
N3—Zn—O1	104.68 (10)
N2—Zn—O1	87.11 (10)
N1—Zn—O1	146.42 (10)
N4—Zn—O2	156.42 (10)
N3—Zn—O2	90.89 (10)
N2—Zn—O2	95.35 (10)
N1—Zn—O2	92.10 (10)
O1—Zn—O2	59.45 (9)

**Table 2:** Hydrogen-bond geometry (Å, °)

*D*—H⋯*A*	*D*—H	H⋯*A*	*D*⋯*A*	*D*—H⋯*A*
O5—H5*A*⋯O8^i^	0.84 (2)	1.95 (2)	2.776 (5)	165 (5)
O5—H5*B*⋯O10	0.84 (4)	1.90 (4)	2.733 (6)	170 (4)
O6—H6*A*⋯O4^ii^	0.85 (4)	2.02 (2)	2.861 (5)	176 (3)
O6—H6*B*⋯O7	0.83 (3)	2.16 (3)	2.985 (5)	170 (3)
O7—H7*A*⋯O5	0.85 (3)	1.89 (3)	2.734 (5)	176 (5)
O7—H7*B*⋯O2^iii^	0.86 (2)	1.97 (2)	2.828 (4)	174 (5)
O8—H8*A*⋯O9	0.85 (3)	1.96 (3)	2.804 (4)	172 (4)
O8—H8*B*⋯O4^ii^	0.85 (3)	1.99 (3)	2.832 (4)	173 (3)
O9—H9*A*⋯O7	0.84 (4)	1.95 (2)	2.789 (4)	175 (4)
O9—H9*B*⋯O3^iv^	0.85 (3)	1.89 (3)	2.736 (4)	173 (4)
O10—H10*A*⋯O3^iv^	0.86 (2)	1.88 (4)	2.732 (4)	171 (4)
O10—H10*B*⋯O1	0.85 (3)	2.11 (3)	2.957 (4)	176 (4)
C2—H2⋯O9^v^	0.93	2.53	3.429 (5)	162
C5—H5⋯O2^vi^	0.93	2.55	3.381 (4)	149
C17—H17⋯O1^vii^	0.93	2.59	3.263 (4)	129
C18—H18⋯O6^iv^	0.93	2.50	3.344 (5)	151
C26—H26*B*⋯*Cg*1^i^	0.97	2.99	3.791 (4)	140
C27—H27*A*⋯*Cg*2^i^	0.97	2.82	3.375 (4)	117

Symmetry codes: (i) 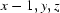; (ii) 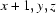; (iii) 
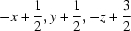
; (iv) 
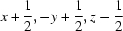
; (v) 
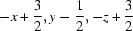
; (vi) 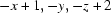; (vii) 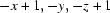. *Cg*1 and *Cg*2 are the centroids of the C16–C19/C23/C24 and C23/C19–C22/N4 rings, respectively.

**Fig. 1.**
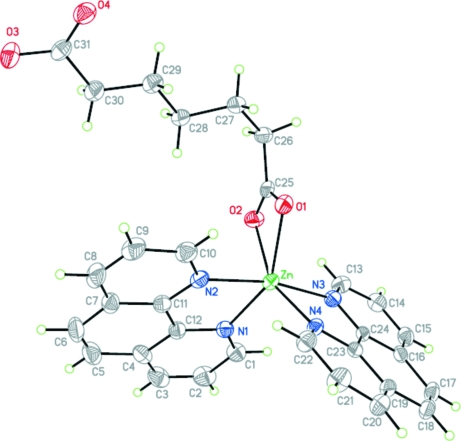
The molecular structure of the ZnII complex, showing 30% probability displacement ellipsoids and the atom-numbering scheme.

**Fig. 2.**
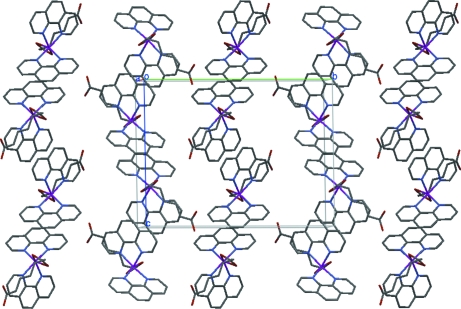
A view of a single layer of (I). H atoms and water molecules have been omitted.

## supplementary crystallographic information

### Comment

Previous investigation on self-assembly of metal ions, hetroaromatic N-donor ligands and pimelate anions exhibits various coordinating modes of pimelate anions. For example, pimelate anion bridges two metal ions in bis-monodentate fashion (Zheng, Lin *et al.*, 2003; Ge & Zheng, 2005; Zheng & Ying, 2004), in chelting/monodentate fashion (Zheng, 2004) and in bis-chelaing fashion (Zheng, Kong *et al.*, 2003). When bridging three metal ions, pimelate anion can offer one carboxylate monoatomically to bridge two metal ions and the other end monodentately to coordinate one metal ion (Zheng *et al.*, 2001; Ge & Zheng, 2005). Furthermore, pimelate anion can bischelate two Cd atoms with one oxygen bonded to additional Cd atom to bridge three metal atoms to form polymeric chains (Zheng *et al.*, 2002). To the best of our knowledge, the title Zn compound represents a new example with pimelate anion coordinating one metal atom in a chelating fashion.

The title compound consists of [Zn(phen)_2_(C_7_H_10_O_4_)] complex and hydrogen bonded H_2_O molecules. As demonstrated in Fig.1, the Zn atom in the complex cation is coordinated by two carboxylato oxygen atoms of one mono-chelating pimelate (C_7_H_10_O_4_)^2-^ anion and four nitrogen atoms of two phenanthroline (phen) ligands to define a considerably distorted octahedral ZnN_4_O_2_ chromophore. Two phen ligands chelating the central Zn atom form V-shaped cleft and the mono-chelating pimelato ligand is twisted at the carbon atom next to the chelating carboxylato end to adopt a *gauche* conformation around the C26—C27 bond. The present complex looks very like the monovalent [Zn(phen)_2_(C_8_H_13_O_4_)]^+^ complex cation found in the earlier-reported [Zn(phen)_2_(C_8_H_13_O_4_)](NO_3_).H_2_O, where the Zn atoms are coordinated by two phen ligands and one hydrogen suberate (C_8_H_13_O_4_)^-^ anion (Wei *et al.*, 2002).

Along [001] direction, the complex are arranged with the clefts orientating alternatively up- and downwards and the symmetry-related phen ligands orientate anti-parallelly to each other and the mean interplanar distance of 3.399 (2) Å suggests that the N-donor ligands are engaged in intercationic π-π stacking interactions. In this sense, the complex cations are, *via*π-π stacking interactions, assembled into one-dimensional chains extending parallel to [001] and careful inspection indicates that the resulting chains are stabilized by intercationic C5—H5···O2 hydrogen bonds. In the (011) plane, the chains are so arranged that the twisted pimelato ligands are located in the clefts of the adjacent chains and the alkyl C—H bonds are directed to the phen plane (C1 to C12) to form C—H···π interactions. According to the above description, supramolecular assembly of the complex cations into two-dimensional layers (Fig. 2) is achieved due to intercationic π-π, C—H···O and C—H···π interactions. The lattice H_2_O molecules are sandwiched between the cationic layers and form hydrogen bonded anionic chains propagating along [100]. The water molecules except the O5 one are hydrogen-bonded to the carboxylate O atoms.

### Experimental

NaOH (2.0 ml, 1 *M*) was dropwise added to a stirred solution of Zn(NO_3_)_2_.6H_2_O (0.295 g, 0.99 mmol) in H_2_O (5.0 ml) to produce white precipitate, which was separated by centrifugation and washed with deionized water for several times. The fresh precipitate was moved to a solution of phenanthroline monohydrate (0.200 g, 1.0 mmol) and pimelic acid (0.162 g, 1.0 mmol) in CH_3_OH/H_2_O (1:1 *v*/*v*; 20 ml), and then NaOH (0.5 ml, 1 *M*) was dropwise added. The resulting suspension was filtered out and the colorless filtrate (pH = 8.57) was allowed to stand at room temperature and slow evaporation for several weeks afforded a mixture of prismatic colorless crystals of [Zn(C_12_H_8_N_2_)_2_(C_7_H_10_O_4_)].6H_2_O and plate-like colorless crystals. The former crystals are stable, and the latter are found to easily deteriorate upon isolation from the mother liquor.

### Refinement

H atoms of water molecules were located in a difference Fourier map, and were refined with distance restraints of O—H = 0.85 (2) and H···H = 1.38 (2) Å, and with *U*_iso_(H) = 1.2*U*_eq_(O). Other H atoms were placed in geometrically idealized positions (C—H = 0.93–0.97 Å) and constrained to ride on their parent atoms with *U*_iso_(H) = 1.2*U*_eq_(C).

### Crystal data

**Table:**

[Zn(C_7_H_10_O_4_)(C_12_H_8_N_2_)_2_]·6H_2_O	*F*_000_ = 1448
*M**_r_* = 692.02	*D*_x_ = 1.425 Mg m^−^^3^
Monoclinic, *P*2_1_/*n*	Melting point: 163 K
Hall symbol: -P 2yn	Mo *K*α radiation λ = 0.71073 Å
*a* = 9.2050 (18) Å	Cell parameters from 25 reflections
*b* = 21.241 (4) Å	θ = 5–12.5º
*c* = 16.598 (3) Å	µ = 0.82 mm^−^^1^
β = 96.48 (3)º	*T* = 296 (2) K
*V* = 3224.6 (11) Å^3^	Block, colourless
*Z* = 4	0.43 × 0.26 × 0.22 mm

### Data collection

**Table:**

Bruker P4 diffractometer	*R*_int_ = 0.043
Radiation source: fine-focus sealed tube	θ_max_ = 27.5º
Monochromator: graphite	θ_min_ = 1.6º
*T* = 296(2) K	*h* = −11→1
θ/2θ scans	*k* = −1→27
Absorption correction: ψ scan(North *et al*., 1968)	*l* = −21→21
*T*_min_ = 0.697, *T*_max_ = 0.834	3 standard reflections
9226 measured reflections	every 97 reflections
7393 independent reflections	intensity decay: no
3956 reflections with *I* > 2σ(*I*)	

### Refinement

**Table:**

Refinement on *F*^2^	Secondary atom site location: difference Fourier map
Least-squares matrix: full	Hydrogen site location: inferred from neighbouring sites
*R*[*F*^2^ > 2σ(*F*^2^)] = 0.059	H atoms treated by a mixture of independent and constrained refinement
*wR*(*F*^2^) = 0.117	*w* = 1/[σ^2^(*F*_o_^2^) + (0.0346*P*)^2^ + 1.0334*P*] where *P* = (*F*_o_^2^ + 2*F*_c_^2^)/3
*S* = 1.01	(Δ/σ)_max_ = 0.001
7393 reflections	Δρ_max_ = 0.34 e Å^−^^3^
453 parameters	Δρ_min_ = −0.29 e Å^−^^3^
18 restraints	Extinction correction: SHELXL97 (Sheldrick, 2008), Fc^*^=kFc[1+0.001xFc^2^λ^3^/sin(2θ)]^-1/4^
Primary atom site location: structure-invariant direct methods	Extinction coefficient: 0.00132 (18)

### Special details

**Table:**

Geometry. All e.s.d.'s (except the e.s.d. in the dihedral angle between two l.s. planes) are estimated using the full covariance matrix. The cell e.s.d.'s are taken into account individually in the estimation of e.s.d.'s in distances, angles and torsion angles; correlations between e.s.d.'s in cell parameters are only used when they are defined by crystal symmetry. An approximate (isotropic) treatment of cell e.s.d.'s is used for estimating e.s.d.'s involving l.s. planes.
Refinement. Refinement of *F*^2^ against ALL reflections. The weighted *R*-factor *wR* and goodness of fit *S* are based on *F*^2^, conventional *R*-factors *R* are based on *F*, with *F* set to zero for negative *F*^2^. The threshold expression of *F*^2^ > σ(*F*^2^) is used only for calculating *R*-factors(gt) *etc*. and is not relevant to the choice of reflections for refinement. *R*-factors based on *F*^2^ are statistically about twice as large as those based on *F*, and *R*- factors based on ALL data will be even larger.

### Fractional atomic coordinates and isotropic or equivalent isotropic displacement parameters (Å^2^)

**Table:**

	*x*	*y*	*z*	*U*_iso_*/*U*_eq_	
Zn	0.45796 (4)	0.04742 (2)	0.72867 (2)	0.03662 (14)	
N1	0.6065 (3)	0.00835 (14)	0.82659 (17)	0.0385 (7)	
N2	0.4468 (3)	0.11526 (14)	0.82373 (17)	0.0396 (7)	
C1	0.6859 (4)	−0.04345 (19)	0.8282 (2)	0.0484 (9)	
H1	0.6865	−0.0662	0.7804	0.058*	
C2	0.7686 (5)	−0.0660 (2)	0.8973 (3)	0.0619 (12)	
H2	0.8243	−0.1024	0.8952	0.074*	
C3	0.7668 (5)	−0.0338 (2)	0.9682 (3)	0.0629 (12)	
H3	0.8202	−0.0486	1.0153	0.075*	
C4	0.6848 (4)	0.02125 (19)	0.9703 (2)	0.0486 (10)	
C5	0.6771 (5)	0.0579 (2)	1.0425 (2)	0.0644 (13)	
H5	0.7280	0.0448	1.0911	0.077*	
C6	0.5972 (5)	0.1109 (2)	1.0404 (2)	0.0654 (13)	
H6	0.5950	0.1339	1.0878	0.079*	
C7	0.5155 (4)	0.13299 (19)	0.9674 (2)	0.0478 (10)	
C8	0.4325 (5)	0.1880 (2)	0.9616 (3)	0.0614 (12)	
H8	0.4282	0.2131	1.0072	0.074*	
C9	0.3580 (5)	0.20512 (19)	0.8897 (3)	0.0575 (11)	
H9	0.3010	0.2414	0.8859	0.069*	
C10	0.3680 (4)	0.16756 (19)	0.8216 (2)	0.0513 (10)	
H10	0.3171	0.1798	0.7725	0.062*	
C11	0.5207 (4)	0.09748 (17)	0.8962 (2)	0.0405 (9)	
C12	0.6062 (4)	0.04080 (17)	0.8974 (2)	0.0394 (9)	
N3	0.5138 (3)	−0.02261 (14)	0.64572 (17)	0.0359 (7)	
N4	0.5801 (3)	0.10061 (13)	0.65101 (16)	0.0339 (7)	
C13	0.4767 (4)	−0.08320 (19)	0.6419 (2)	0.0479 (10)	
H13	0.4083	−0.0977	0.6747	0.058*	
C14	0.5360 (4)	−0.12543 (18)	0.5911 (2)	0.0474 (10)	
H14	0.5051	−0.1671	0.5891	0.057*	
C15	0.6391 (4)	−0.10603 (18)	0.5445 (2)	0.0455 (10)	
H15	0.6815	−0.1345	0.5115	0.055*	
C16	0.6810 (4)	−0.04252 (17)	0.54645 (18)	0.0357 (8)	
C17	0.7875 (4)	−0.01760 (19)	0.4982 (2)	0.0458 (10)	
H17	0.8350	−0.0444	0.4654	0.055*	
C18	0.8192 (4)	0.0444 (2)	0.4999 (2)	0.0451 (9)	
H18	0.8873	0.0599	0.4676	0.054*	
C19	0.7503 (4)	0.08676 (17)	0.5505 (2)	0.0366 (8)	
C20	0.7791 (4)	0.15174 (18)	0.5536 (2)	0.0471 (10)	
H20	0.8472	0.1691	0.5227	0.057*	
C21	0.7055 (5)	0.18921 (19)	0.6029 (2)	0.0542 (11)	
H21	0.7200	0.2326	0.6036	0.065*	
C22	0.6091 (4)	0.16208 (17)	0.6518 (2)	0.0444 (9)	
H22	0.5632	0.1879	0.6865	0.053*	
C23	0.6491 (3)	0.06355 (16)	0.60015 (19)	0.0320 (8)	
C24	0.6144 (3)	−0.00242 (16)	0.59791 (18)	0.0293 (8)	
O1	0.2329 (3)	0.07270 (12)	0.68624 (14)	0.0445 (6)	
O2	0.2732 (3)	−0.01112 (12)	0.76159 (15)	0.0468 (7)	
C25	0.1853 (4)	0.02656 (18)	0.7233 (2)	0.0386 (9)	
C26	0.0227 (4)	0.01661 (18)	0.7220 (2)	0.0451 (10)	
H26A	0.0056	−0.0170	0.7594	0.054*	
H26B	−0.0172	0.0034	0.6680	0.054*	
C27	−0.0573 (4)	0.07532 (18)	0.7449 (2)	0.0463 (10)	
H27A	−0.1616	0.0672	0.7362	0.056*	
H27B	−0.0365	0.1091	0.7086	0.056*	
C28	−0.0188 (4)	0.09761 (17)	0.8318 (2)	0.0427 (9)	
H28A	−0.0387	0.0641	0.8687	0.051*	
H28B	0.0849	0.1070	0.8408	0.051*	
C29	−0.1048 (4)	0.15551 (18)	0.8501 (2)	0.0474 (10)	
H29A	−0.0795	0.1893	0.8149	0.057*	
H29B	−0.2079	0.1466	0.8365	0.057*	
C30	−0.0816 (4)	0.17858 (18)	0.9365 (2)	0.0497 (10)	
H30A	0.0187	0.1929	0.9476	0.060*	
H30B	−0.0934	0.1431	0.9720	0.060*	
C31	−0.1816 (4)	0.23129 (17)	0.9587 (2)	0.0424 (9)	
O3	−0.1667 (3)	0.24772 (12)	1.03240 (15)	0.0561 (7)	
O4	−0.2700 (3)	0.25491 (12)	0.90506 (16)	0.0518 (7)	
O5	0.0621 (4)	0.29022 (19)	0.7220 (3)	0.0979 (13)	
H5A	−0.029 (2)	0.294 (2)	0.722 (3)	0.117*	
H5B	0.082 (5)	0.2586 (18)	0.695 (3)	0.117*	
O6	0.5105 (4)	0.35021 (17)	0.9056 (2)	0.0760 (10)	
H6A	0.577 (4)	0.3225 (18)	0.908 (2)	0.091*	
H6B	0.463 (4)	0.351 (2)	0.8597 (16)	0.091*	
O7	0.3106 (3)	0.36089 (15)	0.7516 (2)	0.0664 (9)	
H7A	0.234 (3)	0.3389 (15)	0.740 (3)	0.080*	
H7B	0.286 (4)	0.3996 (9)	0.744 (3)	0.080*	
O8	0.7737 (3)	0.30601 (17)	0.75204 (18)	0.0672 (9)	
H8A	0.694 (3)	0.312 (2)	0.7220 (19)	0.081*	
H8B	0.754 (4)	0.289 (2)	0.7958 (15)	0.081*	
O9	0.5085 (3)	0.31376 (16)	0.65131 (18)	0.0663 (9)	
H9A	0.453 (4)	0.3297 (19)	0.683 (2)	0.080*	
H9B	0.456 (4)	0.2967 (19)	0.6116 (18)	0.080*	
O10	0.1348 (4)	0.19689 (15)	0.6205 (2)	0.0749 (10)	
H10A	0.196 (4)	0.2109 (18)	0.589 (2)	0.090*	
H10B	0.166 (5)	0.1614 (13)	0.638 (3)	0.090*	

### Atomic displacement parameters (Å^2^)

**Table:**

	*U*^11^	*U*^22^	*U*^33^	*U*^12^	*U*^13^	*U*^23^
Zn	0.0340 (2)	0.0411 (3)	0.0360 (2)	0.0035 (2)	0.00918 (16)	−0.0029 (2)
N1	0.0361 (17)	0.0382 (18)	0.0412 (18)	−0.0007 (15)	0.0050 (14)	−0.0012 (15)
N2	0.0360 (17)	0.0397 (18)	0.0446 (19)	−0.0014 (15)	0.0117 (15)	−0.0034 (15)
C1	0.050 (2)	0.044 (2)	0.050 (2)	0.005 (2)	0.0023 (19)	0.003 (2)
C2	0.055 (3)	0.059 (3)	0.070 (3)	0.017 (2)	−0.001 (2)	0.016 (2)
C3	0.062 (3)	0.069 (3)	0.055 (3)	−0.002 (3)	−0.008 (2)	0.016 (2)
C4	0.049 (2)	0.052 (3)	0.043 (2)	−0.011 (2)	−0.0036 (19)	0.006 (2)
C5	0.071 (3)	0.080 (4)	0.041 (2)	−0.012 (3)	−0.001 (2)	0.003 (2)
C6	0.081 (3)	0.076 (3)	0.040 (3)	−0.023 (3)	0.010 (2)	−0.019 (2)
C7	0.053 (2)	0.048 (3)	0.044 (2)	−0.014 (2)	0.012 (2)	−0.0103 (19)
C8	0.072 (3)	0.050 (3)	0.066 (3)	−0.006 (2)	0.020 (3)	−0.029 (2)
C9	0.056 (3)	0.041 (2)	0.078 (3)	0.006 (2)	0.018 (2)	−0.015 (2)
C10	0.047 (2)	0.048 (3)	0.060 (3)	0.002 (2)	0.010 (2)	−0.006 (2)
C11	0.038 (2)	0.043 (2)	0.041 (2)	−0.0112 (19)	0.0085 (18)	−0.0016 (18)
C12	0.041 (2)	0.040 (2)	0.038 (2)	−0.0066 (19)	0.0083 (17)	0.0002 (18)
N3	0.0355 (17)	0.0338 (17)	0.0385 (17)	0.0038 (14)	0.0044 (14)	−0.0020 (13)
N4	0.0336 (16)	0.0360 (18)	0.0329 (16)	0.0057 (14)	0.0078 (13)	−0.0007 (14)
C13	0.043 (2)	0.049 (3)	0.053 (3)	−0.002 (2)	0.010 (2)	0.004 (2)
C14	0.050 (2)	0.037 (2)	0.054 (2)	0.003 (2)	0.000 (2)	−0.005 (2)
C15	0.051 (2)	0.045 (2)	0.039 (2)	0.010 (2)	0.0009 (19)	−0.0086 (18)
C16	0.0365 (19)	0.044 (2)	0.0256 (17)	0.0073 (19)	−0.0020 (15)	−0.0029 (17)
C17	0.054 (2)	0.053 (3)	0.033 (2)	0.011 (2)	0.0116 (18)	−0.0075 (18)
C18	0.047 (2)	0.058 (3)	0.0324 (19)	0.006 (2)	0.0126 (17)	0.003 (2)
C19	0.035 (2)	0.045 (2)	0.0300 (19)	0.0011 (18)	0.0048 (16)	0.0051 (17)
C20	0.051 (2)	0.047 (2)	0.045 (2)	0.000 (2)	0.0145 (19)	0.005 (2)
C21	0.066 (3)	0.036 (2)	0.063 (3)	−0.006 (2)	0.020 (2)	0.002 (2)
C22	0.050 (2)	0.037 (2)	0.047 (2)	0.0023 (19)	0.0128 (19)	−0.0064 (18)
C23	0.0281 (18)	0.040 (2)	0.0274 (18)	0.0062 (16)	0.0009 (15)	0.0012 (15)
C24	0.0280 (18)	0.036 (2)	0.0229 (17)	0.0040 (16)	0.0006 (14)	−0.0008 (15)
O1	0.0456 (15)	0.0491 (16)	0.0402 (14)	−0.0004 (13)	0.0104 (12)	−0.0003 (13)
O2	0.0367 (14)	0.0506 (17)	0.0530 (16)	0.0073 (13)	0.0044 (12)	−0.0009 (13)
C25	0.038 (2)	0.047 (2)	0.033 (2)	−0.0004 (19)	0.0100 (17)	−0.0141 (18)
C26	0.033 (2)	0.053 (2)	0.050 (2)	−0.0004 (19)	0.0042 (17)	−0.013 (2)
C27	0.037 (2)	0.055 (2)	0.046 (2)	0.0071 (19)	0.0028 (18)	−0.0063 (19)
C28	0.034 (2)	0.051 (2)	0.043 (2)	0.0076 (19)	0.0055 (17)	−0.0003 (19)
C29	0.045 (2)	0.045 (2)	0.053 (2)	0.0041 (19)	0.0102 (19)	−0.003 (2)
C30	0.049 (2)	0.051 (3)	0.049 (2)	0.009 (2)	0.0070 (19)	−0.004 (2)
C31	0.046 (2)	0.032 (2)	0.052 (3)	0.0022 (19)	0.018 (2)	−0.0001 (19)
O3	0.075 (2)	0.0500 (17)	0.0447 (17)	0.0128 (16)	0.0137 (15)	−0.0042 (14)
O4	0.0510 (17)	0.0512 (17)	0.0543 (17)	0.0130 (14)	0.0110 (14)	0.0012 (14)
O5	0.076 (2)	0.083 (3)	0.145 (4)	−0.020 (2)	0.054 (3)	−0.036 (2)
O6	0.073 (2)	0.078 (2)	0.077 (2)	0.0225 (19)	0.0106 (18)	−0.023 (2)
O7	0.0578 (19)	0.060 (2)	0.083 (2)	0.0079 (16)	0.0120 (18)	−0.0120 (19)
O8	0.055 (2)	0.095 (3)	0.0522 (18)	0.0039 (19)	0.0068 (15)	0.0066 (18)
O9	0.0517 (19)	0.083 (2)	0.064 (2)	0.0020 (17)	0.0033 (15)	−0.0208 (17)
O10	0.087 (2)	0.059 (2)	0.085 (2)	0.0016 (19)	0.042 (2)	0.0066 (18)

### Geometric parameters (Å, °)

**Table:**

Zn—N4	2.128 (3)	C17—H17	0.9300
Zn—N3	2.129 (3)	C18—C19	1.427 (5)
Zn—N2	2.148 (3)	C18—H18	0.9300
Zn—N1	2.167 (3)	C19—C23	1.401 (5)
Zn—O1	2.178 (3)	C19—C20	1.405 (5)
Zn—O2	2.224 (3)	C20—C21	1.374 (5)
Zn—C25	2.540 (4)	C20—H20	0.9300
N1—C1	1.320 (4)	C21—C22	1.393 (5)
N1—C12	1.363 (4)	C21—H21	0.9300
N2—C10	1.325 (5)	C22—H22	0.9300
N2—C11	1.367 (4)	C23—C24	1.437 (5)
C1—C2	1.388 (5)	O1—C25	1.262 (4)
C1—H1	0.9300	O2—C25	1.259 (4)
C2—C3	1.363 (6)	C25—C26	1.509 (5)
C2—H2	0.9300	C26—C27	1.519 (5)
C3—C4	1.395 (6)	C26—H26A	0.9700
C3—H3	0.9300	C26—H26B	0.9700
C4—C12	1.400 (5)	C27—C28	1.521 (5)
C4—C5	1.438 (6)	C27—H27A	0.9700
C5—C6	1.341 (6)	C27—H27B	0.9700
C5—H5	0.9300	C28—C29	1.512 (5)
C6—C7	1.431 (6)	C28—H28A	0.9700
C6—H6	0.9300	C28—H28B	0.9700
C7—C8	1.393 (6)	C29—C30	1.507 (5)
C7—C11	1.408 (5)	C29—H29A	0.9700
C8—C9	1.357 (6)	C29—H29B	0.9700
C8—H8	0.9300	C30—C31	1.521 (5)
C9—C10	1.395 (5)	C30—H30A	0.9700
C9—H9	0.9300	C30—H30B	0.9700
C10—H10	0.9300	C31—O4	1.242 (4)
C11—C12	1.437 (5)	C31—O3	1.264 (4)
N3—C13	1.331 (4)	O5—H5A	0.84 (2)
N3—C24	1.355 (4)	O5—H5B	0.84 (4)
N4—C22	1.333 (4)	O6—H6A	0.85 (4)
N4—C23	1.363 (4)	O6—H6B	0.83 (3)
C13—C14	1.385 (5)	O7—H7A	0.85 (3)
C13—H13	0.9300	O7—H7B	0.86 (2)
C14—C15	1.355 (5)	O8—H8A	0.85 (3)
C14—H14	0.9300	O8—H8B	0.85 (3)
C15—C16	1.403 (5)	O9—H9A	0.84 (4)
C15—H15	0.9300	O9—H9B	0.85 (3)
C16—C24	1.396 (4)	O10—H10A	0.86 (4)
C16—C17	1.436 (5)	O10—H10B	0.85 (3)
C17—C18	1.349 (5)		
			
N4—Zn—N3	78.26 (11)	C14—C15—H15	120.4
N4—Zn—N2	99.28 (11)	C16—C15—H15	120.4
N3—Zn—N2	168.21 (11)	C24—C16—C15	117.5 (3)
N4—Zn—N1	109.01 (11)	C24—C16—C17	119.4 (3)
N3—Zn—N1	92.26 (11)	C15—C16—C17	123.0 (3)
N2—Zn—N1	77.55 (11)	C18—C17—C16	120.5 (3)
N4—Zn—O1	102.80 (10)	C18—C17—H17	119.8
N3—Zn—O1	104.68 (10)	C16—C17—H17	119.8
N2—Zn—O1	87.11 (10)	C17—C18—C19	121.3 (3)
N1—Zn—O1	146.42 (10)	C17—C18—H18	119.4
N4—Zn—O2	156.42 (10)	C19—C18—H18	119.4
N3—Zn—O2	90.89 (10)	C23—C19—C20	117.3 (3)
N2—Zn—O2	95.35 (10)	C23—C19—C18	119.6 (3)
N1—Zn—O2	92.10 (10)	C20—C19—C18	123.1 (3)
O1—Zn—O2	59.45 (9)	C21—C20—C19	119.2 (4)
N4—Zn—C25	131.21 (12)	C21—C20—H20	120.4
N3—Zn—C25	99.51 (11)	C19—C20—H20	120.4
N2—Zn—C25	90.80 (11)	C20—C21—C22	119.8 (4)
N1—Zn—C25	119.78 (12)	C20—C21—H21	120.1
O1—Zn—C25	29.77 (10)	C22—C21—H21	120.1
O2—Zn—C25	29.69 (10)	N4—C22—C21	122.6 (3)
C1—N1—C12	117.3 (3)	N4—C22—H22	118.7
C1—N1—Zn	129.5 (3)	C21—C22—H22	118.7
C12—N1—Zn	113.0 (2)	N4—C23—C19	123.3 (3)
C10—N2—C11	118.2 (3)	N4—C23—C24	117.5 (3)
C10—N2—Zn	127.9 (3)	C19—C23—C24	119.2 (3)
C11—N2—Zn	113.6 (2)	N3—C24—C16	122.8 (3)
N1—C1—C2	123.8 (4)	N3—C24—C23	117.3 (3)
N1—C1—H1	118.1	C16—C24—C23	119.9 (3)
C2—C1—H1	118.1	C25—O1—Zn	91.2 (2)
C3—C2—C1	118.8 (4)	C25—O2—Zn	89.2 (2)
C3—C2—H2	120.6	O2—C25—O1	120.1 (3)
C1—C2—H2	120.6	O2—C25—C26	119.9 (3)
C2—C3—C4	120.1 (4)	O1—C25—C26	120.0 (4)
C2—C3—H3	120.0	O2—C25—Zn	61.09 (19)
C4—C3—H3	120.0	O1—C25—Zn	59.01 (18)
C3—C4—C12	117.1 (4)	C26—C25—Zn	177.7 (3)
C3—C4—C5	123.5 (4)	C25—C26—C27	112.9 (3)
C12—C4—C5	119.3 (4)	C25—C26—H26A	109.0
C6—C5—C4	120.7 (4)	C27—C26—H26A	109.0
C6—C5—H5	119.6	C25—C26—H26B	109.0
C4—C5—H5	119.6	C27—C26—H26B	109.0
C5—C6—C7	122.1 (4)	H26A—C26—H26B	107.8
C5—C6—H6	118.9	C26—C27—C28	115.2 (3)
C7—C6—H6	118.9	C26—C27—H27A	108.5
C8—C7—C11	117.5 (4)	C28—C27—H27A	108.5
C8—C7—C6	124.5 (4)	C26—C27—H27B	108.5
C11—C7—C6	118.0 (4)	C28—C27—H27B	108.5
C9—C8—C7	120.3 (4)	H27A—C27—H27B	107.5
C9—C8—H8	119.8	C29—C28—C27	111.8 (3)
C7—C8—H8	119.8	C29—C28—H28A	109.2
C8—C9—C10	119.1 (4)	C27—C28—H28A	109.2
C8—C9—H9	120.5	C29—C28—H28B	109.2
C10—C9—H9	120.5	C27—C28—H28B	109.2
N2—C10—C9	123.0 (4)	H28A—C28—H28B	107.9
N2—C10—H10	118.5	C30—C29—C28	115.8 (3)
C9—C10—H10	118.5	C30—C29—H29A	108.3
N2—C11—C7	122.0 (4)	C28—C29—H29A	108.3
N2—C11—C12	117.5 (3)	C30—C29—H29B	108.3
C7—C11—C12	120.5 (3)	C28—C29—H29B	108.3
N1—C12—C4	122.9 (4)	H29A—C29—H29B	107.4
N1—C12—C11	117.7 (3)	C29—C30—C31	116.5 (3)
C4—C12—C11	119.3 (3)	C29—C30—H30A	108.2
C13—N3—C24	117.8 (3)	C31—C30—H30A	108.2
C13—N3—Zn	128.8 (3)	C29—C30—H30B	108.2
C24—N3—Zn	112.9 (2)	C31—C30—H30B	108.2
C22—N4—C23	117.7 (3)	H30A—C30—H30B	107.3
C22—N4—Zn	129.3 (2)	O4—C31—O3	124.9 (3)
C23—N4—Zn	112.6 (2)	O4—C31—C30	119.2 (3)
N3—C13—C14	122.6 (4)	O3—C31—C30	115.8 (4)
N3—C13—H13	118.7	H5A—O5—H5B	111 (3)
C14—C13—H13	118.7	H6A—O6—H6B	111 (3)
C15—C14—C13	120.0 (4)	H7A—O7—H7B	107 (3)
C15—C14—H14	120.0	H8A—O8—H8B	108 (3)
C13—C14—H14	120.0	H9A—O9—H9B	108 (3)
C14—C15—C16	119.2 (3)	H10A—O10—H10B	107 (3)

### Hydrogen-bond geometry (Å, °)

**Table:**

*D*—H···*A*	*D*—H	H···*A*	*D*···*A*	*D*—H···*A*
O5—H5A···O8^i^	0.84 (2)	1.95 (2)	2.776 (5)	165 (5)
O5—H5B···O10	0.84 (4)	1.90 (4)	2.733 (6)	170 (4)
O6—H6A···O4^ii^	0.85 (4)	2.02 (2)	2.861 (5)	176 (3)
O6—H6B···O7	0.83 (3)	2.16 (3)	2.985 (5)	170 (3)
O7—H7A···O5	0.85 (3)	1.89 (3)	2.734 (5)	176 (5)
O7—H7B···O2^iii^	0.86 (2)	1.97 (2)	2.828 (4)	174 (5)
O8—H8A···O9	0.85 (3)	1.96 (3)	2.804 (4)	172 (4)
O8—H8B···O4^ii^	0.85 (3)	1.99 (3)	2.832 (4)	173 (3)
O9—H9A···O7	0.84 (4)	1.95 (2)	2.789 (4)	175 (4)
O9—H9B···O3^iv^	0.85 (3)	1.89 (3)	2.736 (4)	173 (4)
O10—H10A···O3^iv^	0.86 (2)	1.88 (4)	2.732 (4)	171 (4)
O10—H10B···O1	0.85 (3)	2.11 (3)	2.957 (4)	176 (4)
C2—H2···O9^v^	0.93	2.53	3.429 (5)	162
C5—H5···O2^vi^	0.93	2.55	3.381 (4)	149
C17—H17···O1^vii^	0.93	2.59	3.263 (4)	129
C18—H18···O6^iv^	0.93	2.50	3.344 (5)	151
C26—H26B···Cg1^i^	0.97	2.99	3.791 (4)	140
C27—H27A···Cg2^i^	0.97	2.82	3.375 (4)	117

Symmetry codes: (i) *x*−1, *y*, *z*;  (ii) *x*+1, *y*, *z*; (iii) −*x*+1/2, *y*+1/2, −*z*+3/2; (iv) *x*+1/2, −*y*+1/2, *z*−1/2; (v) −*x*+3/2, *y*−1/2, −*z*+3/2; (vi) −*x*+1, −*y*, −*z*+2; (vii) −*x*+1, −*y*, −*z*+1.
